# Dexamethasone Inhibits TRAIL-Induced Apoptosis through c-FLIP(L) Upregulation and DR5 Downregulation by GSK3β Activation in Cancer Cells

**DOI:** 10.3390/cancers12102901

**Published:** 2020-10-09

**Authors:** Mi-Yeon Jeon, Seon Min Woo, Seung Un Seo, Sang Hyun Kim, Ju-Ock Nam, Shin Kim, Jong-Wook Park, Peter Kubatka, Kyoung-jin Min, Taeg Kyu Kwon

**Affiliations:** 1 Department of Immunology, School of Medicine, Keimyung University, Daegu 42601, Korea; dldkfls2333@naver.com (M.-Y.J.); 406705@kmu.ac.kr (S.M.W.); 407087@kmu.ac.kr (S.U.S.); god98005@dsmc.or.kr (S.K.); j303nih@dsmc.or.kr (J.-W.P.); 2 Department of Pharmacology, School of Medicine, Kyungpook National University, Daegu 41944, Korea; shkim72@knu.ac.kr; 3 Department of Food Science and Biotechnology, Kyungpook National University, Daegu 41566; Korea; namjo@knu.ac.kr; 4 Department of Medical Biology, Jessenius Faculty of Medicine, Comenius University in Bratislava, 03601 Martin, Slovakia; peter.kubatka@uniba.sk; 5 Department of Experimental Carcinogenesis, Division of Oncology, Biomedical Center Martin, Jessenius Faculty of Medicine, Comenius University in Bratislava, 03601 Martin, Slovakia; 6 New Drug Development Center, Daegu-Gyeongbuk Medical Innovation Foundation, Daegu 41061, Korea; 7 Center for Forensic Pharmaceutical Science, Keimyung University, Daegu 42601, Korea

**Keywords:** dexamethasone, TRAIL, DR5, c-FLIP(L), apoptosis, GSK-3β

## Abstract

**Simple Summary:**

Dexamethasone (DEX) is commonly used as immunosuppressive and chemotherapeutic agent. The effects of DEX on cell death is different, depending on cell types and stimuli. Here, we found that DEX inhibited tumor necrosis factor-related apoptosis-inducing ligand (TRAIL)-induced cell death in cancer cells. Upregulation of c-FLIP(L) and downregulation of death receptor 5 (DR5) play a critical role in anti-apoptotic effects of DEX in TRAIL-induced apoptosis. DEX upregulated c-FLIP(L) expression at the transcriptional levels through the GSK-3β signaling pathway. Furthermore, DEX also modulated protein stability of DR5 via the GSK-3β/Cbl axis-mediated ubiquitin–proteasome system. Therefore, DEX-induced GSK3β activation plays a critical role in the modulation of c-FLIP(L) and DR5. This finding suggests that DEX reduced effects of anti-cancer drugs in cancer cells.

**Abstract:**

Dexamethasone (DEX), a synthetic glucocorticoid, is commonly used as immunosuppressive and chemotherapeutic agent. This study was undertaken to investigate the effects of DEX on the tumor necrosis factor-related apoptosis-inducing ligand (TRAIL)-induced apoptosis in cancer cells. We found that upregulation of c-FLIP(L) and downregulation of death receptor 5 (DR5; receptor for TRAIL ligand) contribute to the anti-apoptotic effect of DEX on TRAIL-induced apoptosis. DEX increased c-FLIP(L) expression at the transcriptional levels through the GSK-3β signaling pathway. The pharmacological inhibitor and catalytic mutant of GSK-3β suppressed DEX-induced upregulation of c-FLIP(L) expression. Furthermore, GSK-3β specific inhibitor markedly abolished DEX-mediated reduction of TRAIL-induced apoptosis in human renal cancer cells (Caki-1 and A498), human lung cancer cells (A549), and human breast cancer cells (MDA-MB361). In addition, DEX decreased protein stability of DR5 via GSK-3β-mediated upregulation of Cbl, an E3 ligase of DR5. Knockdown of Cbl by siRNA markedly inhibited DEX-induced DR5 downregulation. Taken together, these results suggest that DEX inhibits TRAIL-mediated apoptosis via GSK-3β-mediated DR5 downregulation and c-FLIP(L) upregulation in cancer cells.

## 1. Introduction

Tumor necrosis factor-related apoptosis-inducing ligand (TRAIL) is a potential target for cancer therapy, owing to its ability to specifically induce apoptosis of tumor cells without affecting noncancerous cells [1]. TRAIL binds death receptors (DRs) and then forms death-inducing signaling complexes (DISC) with adaptor proteins. DISC activate caspases, and consequently induce apoptosis [2]. However, downregulation of the death receptors (DRs) and pro-apoptotic proteins, and upregulation of the anti-apoptotic proteins, such as Bcl-2, Bcl-xL, and c-FLIP(L), leads to resistance to TRAIL-mediated apoptosis.

Glucocorticoids (GCs) regulate multiple physiological processes and are involved in the development and maintenance of many tissues [3,4]. Dexamethasone (DEX), a synthetic glucocorticoid, has been prescribed to treat autoimmune and noninfectious inflammatory diseases [5]. DEX has been reported to inhibit androgen-independent prostate cancer growth via reduction of interlukin-6 production by inhibition of nuclear factor-κB (NF-κB) transcriptional activity [6]. DEX also downregulated amino acid carrier SLC7A5 expression and induced G1/S cell cycle arrest, autophagy, and apoptosis in BeWo choriocarcinoma cells [7]. In contrast, DEX has been shown to protect cell death by apoptotic stimuli in the multiple cells, including human neutrophils, hair cells, human fibroblasts, auditory hair cells, and normal liver cells [8,9,10,11,12]. Furthermore, DEX also reduces cancer cell death by paclitaxel-treatment in breast carcinoma cells.

However, the effect and the molecular mechanism underlying the anti-apoptotic effects of DEX has rarely been demonstrated in cancer cells. The present study was performed to determine the anti-apoptotic effects and molecular mechanism of DEX on TRAIL-treated human carcinoma cells.

## 2. Results

### 2.1. DEX Inhibits TRAIL-Induced Apoptosis

**Figure 1:**
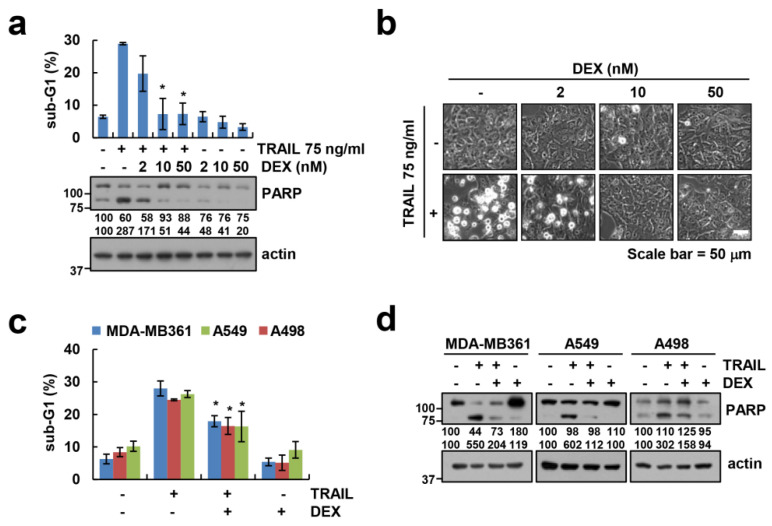
Dexamethasone (DEX) attenuates tumor necrosis factor-related apoptosis-inducing ligand (TRAIL)-induced apoptosis. (**a**–**d**) Caki-1 (**a**,**b**) and indicated cells (**c**,**d**) were treated with DEX for 1 h, and then added with 75 ng/mL TRAIL for 24 h. Apoptosis and protein expression were determined by flow cytometry (**a**,**c**) and Western blotting (**a**,**d**), respectively. Cell morphology was assessed using a light microscope (**b**). Values in the graphs (**a**,**c**) represent mean ± SD of three independent experiments. * *p* < 0.01 compared to TRAIL. Uncropped pictures of the western blot shown in the Supplementary Figure S3.

We examined whether DEX could interfere with anti-cancer drugs-induced apoptosis in human renal carcinoma cells. DEX did not have an effect on DNA damage anti-cancer drugs (doxorubicin and etoposide)-induced and tyrosine kinase inhibitors (sorafenib and gefitinib)-induced apoptosis (Supplementary Figure S1a). Interestingly, DEX markedly inhibited death receptor (TNF-α+cycloheximide (CHX), Fas, and TRAIL)-mediated apoptosis (Supplementary Figure S1b). We focused whether DEX could interfere with TRAIL-induced apoptosis in human renal carcinoma cells. As shown in Figure 1a, the pretreatment with DEX significantly inhibited TRAIL-induced sub-G1 population and cleavage of PARP in a concentration-dependent manner, compared to TRAIL alone. We observed typical apoptotic morphologies, including blebbing, apoptotic bodies, and detachment in TRAIL-treated Caki-1 cells (Figure 1b). When Caki-1 cells were pretreated with 50 nM DEX for 1 h before treatment with 75 ng/mL TRAIL, we did not find apoptotic morphologies (Figure 1b). As shown in Figure 1c,d, DEX treatment attenuated TRAIL-induced sub-G1 population and PARP cleavage in other renal cancer cells (A498), lung cancer cells (A549), and breast cancer cells (MDA-MB361). These data suggested that DEX reduces TRAIL-induced apoptosis in multiple cancer cells.

### 2.2. Effect of DEX on the Expression Level of Apoptosis-Related Proteins

**Figure 2:**
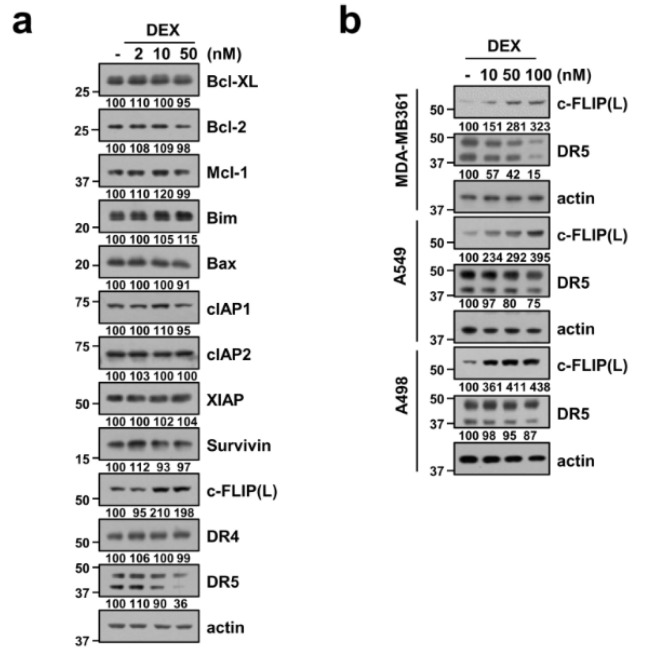
DEX induces c-FLIP(L) upregulation and death receptor 5 (DR5) downregulation. (**a**,**b**) Caki-1 cells (**a**) and indicated cells (**b**) were treated with DEX for 24 h. Protein expression was determined by Western blotting (**a**,**b**). Uncropped pictures of the western blot shown in the Supplementary Figure S4.

To elucidate the molecular mechanisms preventing apoptosis in DEX-treated Caki-1 cells, we investigated the changes of apoptosis-related proteins expression. DEX significantly diminished death receptor 5 (DR5) expression and increased c-FLIP(L) expression in a dose-dependent manner, while other apoptosis-related proteins (Bcl-xL, Bcl-2, Mcl-1, Bim, Bax, cIAP1, cIAP2, XIAP, survivin, and DR4) were not affected by the DEX treatment (Figure 2a). We also examined expression levels of DR5 and c-FLIP(L) protein in other cancer cells. All tested cancer cells revealed a similar expression pattern (Figure 2b). These results indicated that DEX modulates DR5 and c-FLIP(L) expression.

### 2.3. DEX-Induced c-FLIP(L) Upregulation Reduces TRAIL Sensitivity

**Figure 3:**
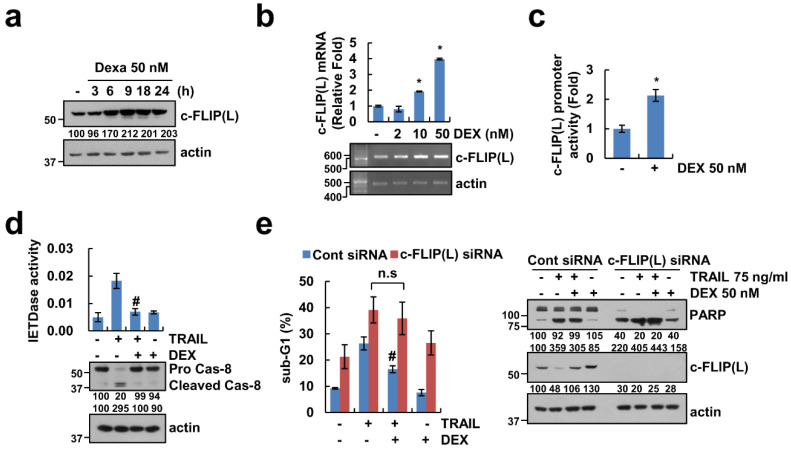
Upregulation of c-FLIP(L) is associated with an anti-apoptotic effect of DEX. (**a**,**b**) Caki-1 cells were treated with 50 nM DEX for the indicated time periods (**a**) or indicated concentrations of DEX for 24 h (**b**). (**c**) Caki-1 cells were treated with 50 nM DEX for 24 h. The luciferase activity was analyzed. (**d**) Caki-1 cells were treated with DEX for 1 h, and then added with 75 ng/mL TRAIL for 24 h. IETDase (caspase-8) activity was examined. (**e**) Caki-1 cells were transiently transfected with control (Cont) siRNA and c-FLIP(L) siRNA, and then treated with 75 ng/mL TRAIL in the absence or presence of 50 nM DEX for 24 h. Protein expression, mRNA expression, and apoptosis were determined by Western blotting (**a**,**d**,**e**), RT or qPCR (**b**), and flow cytometry (**e**), respectively. Values in the graphs (**b**–**e**) represent mean ± SD of three independent experiments. * *p* < 0.01 compared to control. # *p* < 0.01 compared to TRAIL. n.s = no significance. Uncropped pictures of the western blot shown in the Supplementary Figure S5.

Levels of c-FLIP(L) play a major role in TRAIL-mediated apoptosis [13]. Therefore, we investigated whether upregulation of c-FLIP(L) by DEX has a critical role in the reduction of TRAIL sensitivity. As shown in Figure 3a, c-FLIP(L) protein levels were increased by DEX after 6 h. To explore the effect of DEX on c-FLIP(L) expression at the transcriptional level, we checked the c-FLIP(L) mRNA level and promoter activity. As shown in Figure 3b, DEX markedly induced c-FLIP(L) mRNA expression in a dose-dependent manner. Furthermore, DEX treatment increased the promoter activity of c-FLIP(L) in Caki-1 transfected with the c-FLIP(L) promoter construct (Figure 3c). Because c-FLIP(L) is an endogenous inhibitor of caspase-8, we checked activation and cleavage form of caspase-8 by DEX treatment. As expected, DEX abolished caspase-8 activation and cleavege in TRAIL-treated cells (Figure 3d). Next, to investigate the role of c-FLIP(L) upregulation in anti-apoptotic effect of DEX, Caki-1 cells were transfected with c-FLIP(L) siRNA. We found that knockdown of c-FLIP(L) by siRNA alone increased apoptosis, but diminished the inhibitory effect of DEX in TRAIL-treated cells (Figure 3e). These results demonstrate that upregulation of c-FLIP(L) is associated with the inhibitory effect of DEX in TRAIL-induced apoptosis.

### 2.4. GSK-3β Plays a Critical Role on Upregulation of c-FLIP(L) Expression by DEX Treatment

Since multiple kinases are involved in the modulation of c-FLIP(L) expression [14,15,16], we elucidated the signaling pathways capable of modulation of c-FLIP(L) expression by DEX. First, we used MAPKs inhibitors (PD98059 (MEK inhibitor), SB203580 (p38 MAPK inhibitor), and SP600125 (JNK inhibitor)) and a JAK/STAT inhibitor (AG-490). We found MAPKs inhibitors and the JAK/STAT inhibitor did not affect c-FLIP(L) expression in DEX-treated cells (Supplementary Figure S2a and Supplementary Figure S9). In addition, DEX-induced c-FLIP(L) expression was not changed by treatment with the AMPK inhibitor (Compound C), mTOR kinase inhibitors (PP242, NVP-BEZ235, and rapamycin), and the NF-κB inhibitor (BAY11-7082) (Supplementary Figure S2b and Supplementary Figure S9).

**Figure 4:**
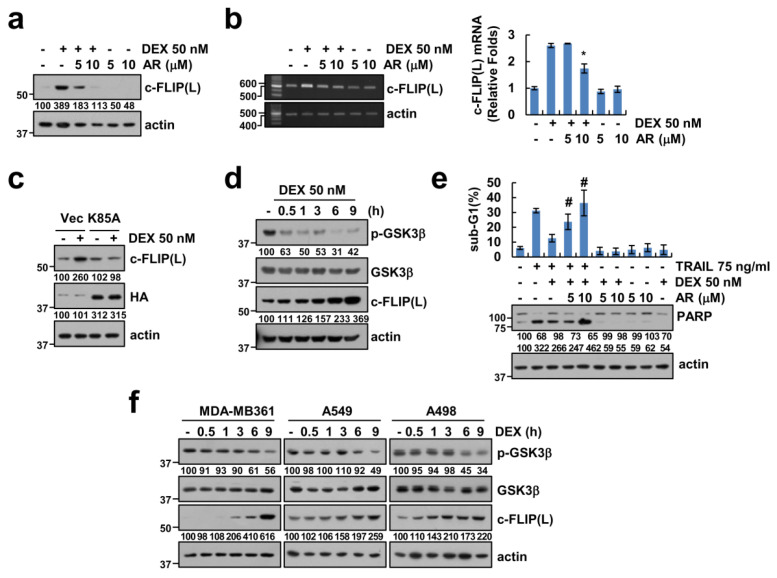
GSK-3β plays a critical role in DEX-induced c-FLIP upregulation. (**a**,**b**) Caki-1 cells were pretreated with a GSK-3β inhibitor (AR-A014418) for 1 h, and then treated with 50 nM DEX for 24 h. (**c**,**d**) Caki-1 cells were treated with 50 nM DEX for 24 h (**c**) or the indicated time periods (**d**). (**e**) Caki-1 cells were pretreated with a GSK-3β inhibitor (AR-A014418) for 1 h, and then added with 75 ng/mL TRAIL in the presence or absence of 50 nM DEX. (**f**) Cells were treated with 50 nM DEX for the indicated time periods. Apoptosis, protein, and mRNA expression were determined by flow cytometry (**e**), Western blotting (**a**,**c**–**f**), PCR, and qPCR (**b**), respectively. Values in the graphs (**b**,**e**) represent mean ± SD of three independent experiments. * *p* < 0.01 compared to DEX. # *p* < 0.01 compared to TRAIL + DEX. Uncropped pictures of the western blot shown in the Supplementary Figure S6.

Previous studies reported that DEX activates glycogen synthase kinase-3β (GSK-3β) pathway [17,18]. Therefore, we determined whether the GSK-3β pathway is involved in DEX-induced c-FLIP(L) upregulation. As shown in Figure 4a,b, the GSK-3β specific inhibitor, AR-A014418, inhibited c-FLIP(L) protein and mRNA expression in DEX-treated cells. To verify the functional role of GSK-3β in upregulation of c-FLIP(L) by DEX, we used constructs with a catalytically inactive mutant (K85A) of GSK-3β. Mutant GSK-3β markedly impeded c-FLIP(L) upregulation by DEX in Caki-1 cells (Figure 4c). Next, we determined whether the GSK-3β pathway is activated in DEX-treated Caki-1 cells. As shown in Figure 4d, we found that the DEX treatment did not change total protein levels of GSK-3β. However, phosphorylation of GSK-3β at Ser-9 was significantly reduced in DEX-treated Caki-1 cells, suggesting that the GSK-3β pathway is activated in Caki-1 cells by the DEX treatment. Next, we investigated whether GSK-3β activation is involved in the inhibition of TRAIL-induced apoptosis by DEX. As expected, AR-A014418 abolished the DEX-mediated reduction in the TRAIL-induced sub-G1 population and cleavage of PARP in a concentration-dependent manner (Figure 4e). In addition, we also examined the effect of DEX on GSK-3β activation and upregulation of c-FLIP(L) protein in other cancer cells. DEX induced dephosphorylation of GSK-3β and upregulation of c-FLIP(L) in all tested cancer cells (Figure 4f). These results indicated that DEX attenuates TRAIL-induced apoptosis via GSK-3β-mediated upregulation of c-FLIP(L) expression.

### 2.5. DEX Treatment Decreases the Expression of DR5

**Figure 5:**
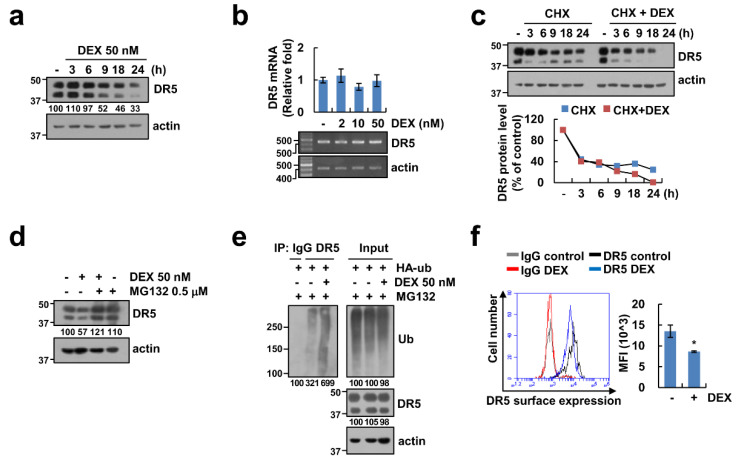
DEX reduces DR5 protein stability. (**a**,**b**) Caki-1 cells were treated with 50 nM DEX for the indicated time periods (**a**) or indicated concentrations of DEX for 24 h (**b**). (**c**) Cells were treated with 20 μg/mL cycloheximide (CHX), in presence or absence of 50 nM DEX for the indicated time points. Band intensity of DR5 was analyzed using ImageJ. (**d**) Caki-1 cells were pretreated with 0.5 μM MG132, and then treated with 50 nM DEX for 24 h. (**e**) DR5 ubiquitination was detected by Western blotting. (**f**) Caki-1 cells were treated with 50 nM DEX for 24 h and measured DR5 expression on the cell surface. Protein and mRNA levels were analyzed by Western blotting (**a**,**c**–**e**) and RT-PCR or qPCR (**b**), respectively. Values in the graphs (**b**,**f**) represent mean ± SD of three independent experiments. * *p* < 0.01 compared to control. Uncropped pictures of the western blot shown in the Supplementary Figure S7.

We confirmed the change of DR5 proteins in DEX-treated Caki-1 cells by Western blot analysis. As shown in Figure 5a, the expression levels of DR5 protein were downregulated by DEX in a time dependent manner. To explore the modulation of DEX-mediated DR5 expression at the transcriptional levels, we checked the DR5 mRNA level. However, DEX did not alter the DR5 mRNA level (Figure 5b). Next, we investigated the impact of DEX on DR5 protein stability using the CHX. Combined treatment with CHX and DEX decreased the DR5 protein level more than compared to CHX alone (Figure 5c). MG132, a proteasome inhibitor, reversed DEX-induced DR5 downregulation (Figure 5d), and DEX dramatically enhanced ubiquitination of DR5 (Figure 5e). Furthermore, DEX decreased DR5 expression on the cellular surface (Figure 5f). Therefore, these data indicated that DEX induces downregulation of DR5 expression at the post-translational levels, resulting in a decrease of DR5 expression on the surface.

### 2.6. Upregulation of Cbl Plays a Critical Role in DEX-Induced DR5 Downregulation

**Figure 6:**
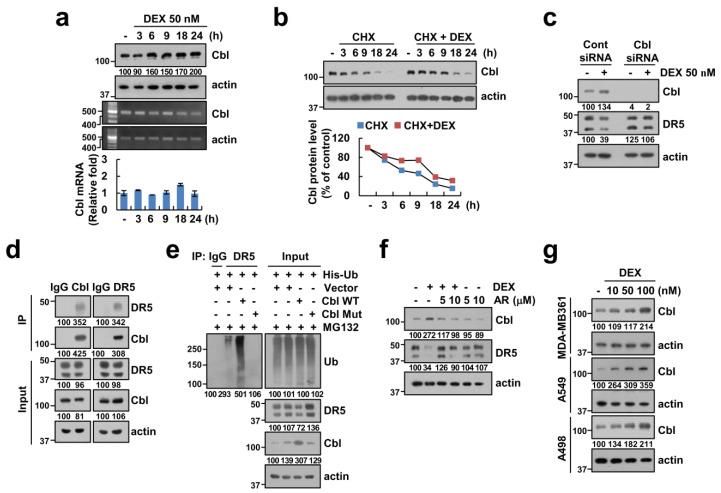
DEX increases ubiquitination of DR5 via upregulation of Cbl expression. (**a**) Caki-1 cells were treated with 50 nM DEX for the indicated time periods. (**b**) Cells were treated with 20 μg/mL CHX in presence or absence of 50 nM DEX for the indicated time points. (**c**) Caki-1 cells were treated with 50 nM DEX for 24 h. (**d**) The interaction with Cbl and DR5 was indicated by immunoprecipitation (IP) assay. (**e**) Caki cells were treated with 0.5 μM MG132 for 12 h. (**f**) Caki-1 cells were pretreated with GSK-3β inhibitor (AR-A014418) for 1 h, and then treated with 50 nM DEX for 24 h. (**g**) We used the same lysates in Figure 2b. Protein and mRNA expression were measured by Western blotting (**a**–**g**) and RT-PCR or qPCR (**a**), respectively. Band intensity of Cbl was analyzed using ImageJ. Values in the graphs (**a**) represent mean ± SD of three independent experiments. Uncropped pictures of the western blot shown in the Supplementary Figure S8.

Previous studies reported that the ubiquitin/deubiquitin–proteasome pathway contributes to the modulation of DR5 expression [19,20]. Therefore, we examined protein expression levels of Cbl E3 ligase, which could regulate the protein stability of DR5, in the DEX-treated cells. As shown in Figure 6a, DEX increased Cbl protein expression, but not Cbl mRNA. We also found that DEX-induced upregulation of Cbl expression is related to Cbl stabilization (Figure 6b). Therefore, we further examined whether Cbl can regulate DEX-mediated DR5 downregulation. As shown in Figure 6c, knockdown of Cbl by siRNA inhibited DEX-mediated DR5 downregulation in Caki-1 cells. Next, we examined whether Cbl directly can regulate DR5 ubiquitination in cells. We found that Cbl E3 ligase reciprocally bound to DR5 (Figure 6d). Cbl markedly induced ubiquitination of DR5, but catalytic mutant Cbl dramatically inhibited DR5 ubiquitination in the transfection of cells with HA-Ub expressing vector (Figure 6e). Interestingly, GSK-3β specific inhibitor, AR-A014418, inhibited DEX-induced Cbl upregulation and DR5 downregulation (Figure 6f). DEX also induced Cbl expression in other renal carcinoma and other type cancer cells (Figure 6g). These data suggested that DEX reduces the protein stability of DR5 via GSK-3β-mediated Cbl upregulation.

## 3. Discussion

**Figure 7:**
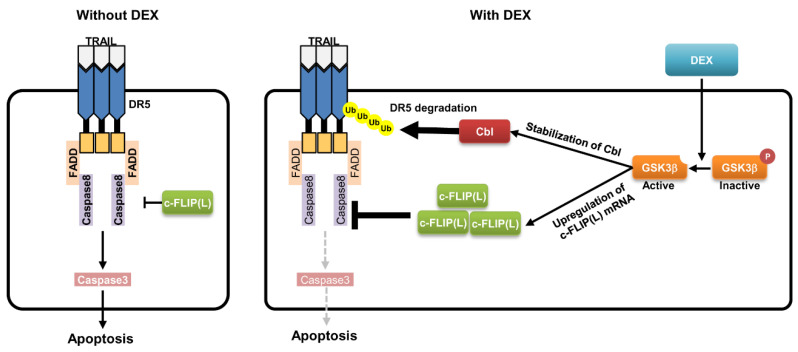
The scheme indicating the inhibitory mechanism of DEX in TRAIL-induced apoptosis.

In this study, we found that DEX markedly inhibited TRAIL-induced apoptosis in cancer cells, and induces downregulation of DR5 and upregulation of c-FLIP(L). DEX increased c-FLIP(L) expression at the transcriptional level via activation of the GSK-3β signal pathway (Figure 7). In addition, upregulation of Cbl E3 ligase by DEX treatment is associated with DR5 downregulation via the ubiquitin–proteasome pathway. Our data suggested that DEX could attenuate TRAIL-induced apoptosis via the modulation of DR5 and c-FLIP(L) expression, which are important for DISC formation.

Glucocorticoids play a critical role in the development and physiological control in animals. The synthetic glucocorticoid DEX induced irreversible G1 arrest and death of lymphoid leukemia in high concentration (10^-6^ M) [21]. Recently, Shaokun et al. reported the biphasic effect of DEX on the viability of osteoblast: a high dose of DEX (≥10^-6^ M) accelerated cell apoptosis, while a low dose of DEX (10^-8^ M) increased cell viability [22]. The anti-apoptotic effect of DEX has been reported to be due to the increased expression of anti-apoptotic Bcl-2 and Bcl-xL proteins in human and rat hepatocytes [23]. However, DEX did not change anti-apoptotic Bcl-2 family proteins in our system (Figure 2a). Therefore, the modulatory effect of DEX on cell death is dependent on the DEX concentrations and cell context. In our study, one of the mechanisms of DEX-mediated TRAIL desensitization is downregulation of DR5 expression, which is critical for TRAIL-mediated apoptosis [24]. As shown in Figure 2a,b, DEX decreased DR5 protein expression. Multiple studies have investigated the transcriptional and post-transcriptional regulation of DR5 by a variety of transcription factors and ubiquitin–proteasome pathway, respectively [25]. Since DEX did not alter DR5 mRNA expression levels (Figure 5b), we investigated other modulatory mechanisms. Recently, our and another group reported that Cbl binds to DR5 and induces degradation of DR5 in TRAIL-treated cells [20,26]. As shown in Figure 6a,c, DEX treatment markedly increased Cbl expression, and downregulation of Cbl by siRNA abolished DEX-induced DR5 downregulation. Our results demonstrated that DEX modulates DR5 expression via upregulation of Cbl expression. Studies of Cbl expression regulatory mechanisms are not yet known. Rafig et al. reported that hypertrophic agonists and inflammatory cytokines increases Cbl protein expression in neonatal rat cardiomyocytes, but detail mechanism was not determined [27]. Arsenic sulfide (As4S4) also increases Cbl expression in chronic myelogenous leukemia. They reported that arsenic directly targets Cbl, and then inhibits self-ubiquitination/degradation of Cbl [28]. In our study, we found that GSK3β plays a critical role in upregulation of Cbl expression, and this is the novel mechanism. We need further investigation to identify the accurate mechanism of GSK3β-mediated upregulation of Cbl.

c-FLIP(L) is a key regulator of the anti-apoptotic function and its association with death receptor-mediated apoptosis has been widely studied [13]. c-FLIP(L) protein expression is regulated by various events such as the transcriptional, post-transcriptional, and ubiquitin–proteasome system [15,29,30]. Interestingly, DEX (300 μM) downregulated c-FLIP(L) protein independent of mRNA expression in acute lymphoblastic leukemia [31]. However, in our study, DEX-induced c-FLIP(L) downregulation was modulated by transcriptional regulation (Figure 3b,c). Oh et al. reported that DEX protected TNF-α and anti-Fas antibody-induced apoptosis via upregulation of c-FLIP(L) in primary cultured hepatocytes [32]. However, their study was focused on normal primary hepatocytes and they did not investigate detail molecular mechanism of DEX-induced c-FLIP(L) upregulation. The NF-κB transcriptional factor is a major role in c-FLIP(L) transcriptional regulation [33]. Methylglyoxal and small-molecules survivin inhibitor (YM155) suppressed c-FLIP(L) mRNA expression through inhibition of NF-κB transcriptional activity [34,35]. However, DEX-induced upregulation of c-FLIP(L) was not inhibited by treatment with NF-κB inhibitor (Supplementary Figure S2b). As shown in Figure 4a,c, the pharmacological inhibitor and catalytic mutant of GSK-3β suppressed DEX-induced c-FLIP(L) expression. Therefore, GSK-3β might be a possible major signal pathway to DEX-induced c-FLIP(L) upregulation.

It is interesting to note that DEX induced c-FLIP(L) upregulation and Cbl-dependent DR5 downregulation through activation of the GSK-3β signal pathway. These findings indicate that DEX has the anti-cancer effect of TRAIL-induced apoptosis, thus providing a novel molecular target for DEX-mediated inhibitory effect on TRAIL-induced apoptosis in cancer cells.

## 4. Materials and Methods

### 4.1. Cell Cultures and Materials

Human renal carcinoma (Caki-1 and A498), human lung cancer (A549), and human breast cancer (MDA-MB361) were procured from American Type Culture Collection (Manassas, VA, USA). Human recombinant TRAIL, zVAD-fmk, and anti-survivin were provided by the R&D system (Minneapolis, MN, USA). MG132, PD98059, AG-490, compound C, and NVP-BEZ235 were supplied from Calbiochem (San Diego, CA, USA). Dexamethasone, cycloheximide, AR-A014418, PP242, BAY11-7082, rapamycin, and anti-actin were provided from Sigma Chemical Co. (St. Louis, MO, USA). Anti-PARP, anti-Bcl-xL, anti-DR5, anti-cIAP1, anti-caspase-8, anti-phospho-GSK3β, and anti-GSK3β were provided by Cell Signaling Technology (Beverly, MA, USA). Anti-Bim, anti-Bax, and anti-XIAP were obtained from BD Biosciences (San Jose, CA, USA). Anti-Mcl-1, anti-Bcl-2, anti-cIAP2, and anti-Cbl were purchased from Santa Cruz Biotechnology (St. Louis, MO, USA). SB203580, SP600125, and anti-c-FLIP(L) were obtained from Enzo Life Sciences (San Diego, CA, USA). Anti-DR4 were obtained from Abcam (Cambridge, MA, USA). pCMV-Myc-Cbl plasmid was a gift from Dr. S. J. Kim (CHA University, Korea). GSK3betaS9A (1016) was a gift from Scott Friedman (Addgene plasmid # 49492; http://n2t.net/addgene:49492; RRID: Addgene_49492) [36].

### 4.2. Flow Cytometry Analysis

To analyze apoptosis, cells were harvested and fixed with 95% ethanol for at least 1 h at 4 °C. Next, cells were incubated in 1.12% sodium citrate buffer containing RNase at 37 °C for 30 min, added to 50 μg/mL propidium iodide, and analyzed using the BD Accuri™ C6 flow cytometer (BD Biosciences, San Jose, CA, USA).

### 4.3. Western Blotting

Cells were lysed in RIPA lysis buffer (20 mM HEPES and 0.5% Triton X-100, pH 7.6) and supernatant fractions were collected. Proteins were separated by SDS-PAGE and transferred to the nitrocellulose membranes (GE Healthcare Life Science, Pittsburgh, PO, USA). Incubated specific antibodies and bands were detected using the Immobilon Western Chemiluminescent HRP Substrate (EMD Millipore, Darmstadt, Germany).

### 4.4. IETDse (Caspase-8) Activity

To measure IETDase activity, cells were harvested and incubated with reaction buffer containing acetyl-Ile-Glu-Thr-Asp-p-nitroanilide (Ac-IETD-pNA) substrate for 2 h at 37 °C. Thereafter, the absorbance at 405 nm was measured with a spectrophotometer.

### 4.5. Reverse Transcription-Polymerase Chain Reaction (RT-PCR) and Quantitative PCR (qPCR)

To isolate the total RNA, we used the TriZol reagent (Life Technologies, Gaithersburg, MD, USA) and obtained cDNA using M-MLV reverse transcriptase (Gibco-BRL, Gaithersburg, MD, USA). For PCR, we used Blend Taq DNA polymerase (Toyobo, Osaka, Japan) with primers targeting DR5, c-FLIP(L), and actin as mentioned in our previous studies [37]. For qPCR, we utilized the SYBR Fast qPCR Mix (Takara Bio Inc., Shiga, Japan) and reactions were performed on the Thermal Cycler Dice^®^ Real Time System III (Takara Bio Inc., Shiga, Japan). The following primers were used for the amplification of DR5, c-FLIP(L), and actin as described as our previous study [37]. We used actin as a reference gene to calculate the threshold cycle number (Ct) of the DR5 gene and reported the delta-delta Ct values of the genes.

### 4.6. Transfection

To measure c-FLIP(L) luciferase activity, we transfected c-FLIP(L) promoter-constructs into the cells using Lipofectamine™2000 (Invitrogen, Carlsbad, CA, USA). Next, cells were collected and harvested in lysis buffer (25 mM Tris-phosphate pH 7.8, 2 mM EDTA, 1% Triton X-100, and 10% glycerol). The supernatants were used to measure the luciferase activity according to the manufacturer’s instructions (Promega, Madison, WI, USA). For knockdown of the gene, Caki-1 cells were transfected with the control siRNA (Bioneer, Daejeon, Korea), c-FLIP(L) siRNA, and Cbl siRNA (Santa Cruz Biotechnology, St. Louis, MO, USA) using the Lipofectamine® RNAiMAX Reagent (Invitrogen, Carlsbad, CA, USA) [38].

### 4.7. Detection of DR5 on Cell Surface

To examine DR5 expression on the cell surface, cells were incubated with DR5-phycoerythrin (Abcam, Cambridge, MA, USA) in PBS including 10% FCS and 1% sodium azide. We analyzed the surface expression of DR5 using flow cytometry as described in our previous study [37].

### 4.8. Immunoprecipitation

Cells were collected, washed with PBS, lysed with RIPA lysis buffer containing 10 mM nethylmaleimide (NEM) (EMD Millipore, Darmstadt, Germany) and 1 mM PMSF, and then sonicated for protein extraction in ice. After sonication, cell lysates were centrifuged at 13,000 × g for 15 min at 4 °C. The supernatants were incubated with 1 μg of anti-DR5 or anti-Cbl antibody overnight at 4 °C, and then attached to Protein G agarose bead using the rotator at 4 °C for 2 h. Cell lysates were washed and boiled in 2× sample buffer for 10 min. Protein–protein interactions were checked by Western blotting.

### 4.9. Ubiquitination Assay

The assay was performed as described in our previous study [39]. Cells were transfected with HA-tagged ubiquitin (HA-Ub) and treated with MG132 for 12 h. Immunoprecipitation was performed using the anti-DR5, and the ubiquitination of endogenous DR5 was checked using HRP-conjugated anti-Ub under denaturing conditions.

### 4.10. Statistical Analysis

The data were analyzed using a one-way ANOVA and post-hoc comparisons (Student–Newman–Keuls) using the Statistical Package for Social Sciences 22.0 software (SPSS Inc.; Chicago, IL, USA).

## 5. Conclusions

Our data supports that DR5 downregulation and c-FLIP(L) upregulation are one potent mediator of antagonizing effects of DEX in TRAIL-induced apoptosis. Therefore, targeting the c-FLIP and DR5 could be a useful treatment strategy to overcome antagonizing effects of DEX.

## Supplementary Materials

The following are available online at https://www.mdpi.com/2072-6694/12/10/2901/s1, Figure S1: Effect of DEX on anti-cancer drugs-induced cell death. Figure S2: Effect of various multiple kinases inhibitors on DEX-mediated c-FLIP(L) upregulation. Figure S3: Uncropped image of Western blot for Figure 1. Figure S4: Uncropped image of Western blot for Figure 2. Figure S5: Uncropped image of Western blot for Figure 3. Figure S6: Uncropped image of Western blot for Figure 4. Figure S7: Uncropped image of Western blot for Figure 5. Figure S8: Uncropped image of Western blot for Figure 6. Figure S9: Uncropped image of Western blot for Figure S2.

## References

[1] Hao L., Zhao Y., Li Z.G., He H.G., Liang Q., Zhang Z.G., Shi Z.D., Zhang P.Y., Han C.H. (2017). Tumor necrosis factor-related apoptosis-inducing ligand inhibits proliferation and induces apoptosis of prostate and bladder cancer cells. Oncol. Lett..

[2] Wang S. (2008). The promise of cancer therapeutics targeting the TNF-related apoptosis-inducing ligand and TRAIL receptor pathway. Oncogene.

[3] Berger S.A., Cole T.J., Schmid W., Schütz G. (1996). Molecular genetic analysis of glucocorticoid and mineralocorticoid signaling in development and physiological processes. Steroids.

[4] Wintermantel T.M., Bock D., Fleig V., Greiner E.F., Schütz G. (2005). The epithelial glucocorticoid receptor is required for the normal timing of cell proliferation during mammary lobuloalveolar development but is dispensable for milk production. Mol. Endocrinol..

[5] Flammer J.R., Rogatsky I. (2011). Minireview: Glucocorticoids in autoimmunity: Unexpected targets and mechanisms. Mol. Endocrinol. (Baltim. Md.).

[6] Nishimura K., Nonomura N., Satoh E., Harada Y., Nakayama M., Tokizane T., Fukui T., Ono Y., Inoue H., Shin M. (2001). Potential mechanism for the effects of dexamethasone on growth of androgen-independent prostate cancer. J. Natl. Cancer Inst..

[7] He B., Zhang N., Zhao R. (2016). Dexamethasone downregulates SLC7A5 expression and promotes cell cycle arrest, autophagy and apoptosis in BeWo cells. J. Cell. Physiol..

[8] Nieuwenhuis B., Lüth A., Kleuser B. (2010). Dexamethasone protects human fibroblasts from apoptosis via an S1P3-receptor subtype dependent activation of PKB/Akt and Bcl XL. Pharmacol. Res..

[9] Haake S.M., Dinh C.T., Chen S., Eshraghi A.A., Van De Water T.R. (2009). Dexamethasone protects auditory hair cells against TNFalpha-initiated apoptosis via activation of PI3K/Akt and NFkappaB signaling. Hear. Res..

[10] Zhao B., Xie G.J., Li R.F., Chen Q., Zhang X.Q. (2015). Dexamethasone protects normal human liver cells from apoptosis induced by tumor necrosis factor-related apoptosis-inducing ligand by upregulating the expression of P-glycoproteins. Mol. Med. Rep..

[11] Dinh C.T., Chen S., Bas E., Dinh J., Goncalves S., Telischi F., Angeli S., Eshraghi A.A., Van De Water T. (2015). Dexamethasone protects against apoptotic cell death of cisplatin-exposed auditory hair cells in vitro. Otol. Neurotol..

[12] Crozier M., Porter L.A. (2015). Paclitaxel-induced transcriptional regulation of Fas signaling pathway is antagonized by dexamethasone. Breast Cancer Res. Treat..

[13] Geserick P., Drewniok C., Hupe M., Haas T.L., Diessenbacher P., Sprick M.R., Schon M.P., Henkler F., Gollnick H., Walczak H. (2008). Suppression of cFLIP is sufficient to sensitize human melanoma cells to TRAIL-and CD95L-mediated apoptosis. Oncogene.

[14] Panka D.J., Mano T., Suhara T., Walsh K., Mier J.W. (2001). Phosphatidylinositol 3-kinase/Akt activity regulates c-FLIP(L) expression in tumor cells. J. Biol. Chem..

[15] Micheau O., Lens S., Gaide O., Alevizopoulos K., Tschopp J. (2001). NF-kappaB signals induce the expression of c-FLIP(L). Mol. Cell. Biol..

[16] Seidelin J.B., Coskun M., Vainer B., Riis L., Soendergaard C., Nielsen O.H. (2013). ERK controls epithelial cell death receptor signalling and cellular FLICE-like inhibitory protein (c-FLIP(L)) in ulcerative colitis. J. Mol. Med. (Berl.).

[17] Spokoini R., Kfir-Erenfeld S., Yefenof E., Sionov R.V. (2010). Glycogen synthase kinase-3 plays a central role in mediating glucocorticoid-induced apoptosis. Mol. Endocrinol..

[18] Ma Z., Zhong Z., Zheng Z., Shi X.M., Zhang W. (2014). Inhibition of glycogen synthase kinase-3beta attenuates glucocorticoid-induced suppression of myogenic differentiation in vitro. PLoS ONE.

[19] Oh Y.T., Deng L., Deng J., Sun S.Y. (2017). The proteasome deubiquitinase inhibitor b-AP15 enhances DR5 activation-induced apoptosis through stabilizing DR5. Sci. Rep..

[20] Song J.J., Szczepanski M.J., Kim S.Y., Kim J.H., An J.Y., Kwon Y.T., Alcala M.A., Bartlett D.L., Lee Y.J. (2010). c-Cbl-mediated degradation of TRAIL receptors is responsible for the development of the early phase of TRAIL resistance. Cell. Signal..

[21] Harmon J.M., Norman M.R., Fowlkes B.J., Thompson E.B. (1979). Dexamethasone induces irreversible G1 arrest and death of a human lymphoid cell line. J. Cell. Physiol..

[22] Zhang S., Liu Y., Liang Q. (2018). Low-dose dexamethasone affects osteoblast viability by inducing autophagy via intracellular ROS. Mol. Med. Rep..

[23] Bailly-Maitre B., de Sousa G., Boulukos K., Gugenheim J., Rahmani R. (2001). Dexamethasone inhibits spontaneous apoptosis in primary cultures of human and rat hepatocytes via Bcl-2 and Bcl-xL induction. Cell Death Differ..

[24] Van Roosmalen I.A., Quax W.J., Kruyt F.A. (2014). Two death-inducing human TRAIL receptors to target in cancer: Similar or distinct regulation and function?. Biochem. Pharmacol..

[25] Min K.J., Woo S.M., Shahriyar S.A., Kwon T.K. (2019). Elucidation for modulation of death receptor (DR) 5 to strengthen apoptotic signals in cancer cells. Arch. Pharm. Res..

[26] Park E.J., Min K.J., Choi K.S., Kubatka P., Kruzliak P., Kim D.E., Kwon T.K. (2016). Chloroquine enhances TRAIL-mediated apoptosis through up-regulation of DR5 by stabilization of mRNA and protein in cancer cells. Sci. Rep..

[27] Rafiq K., Kolpakov M.A., Seqqat R., Guo J., Guo X., Qi Z., Yu D., Mohapatra B., Zutshi N., An W. (2014). c-Cbl inhibition improves cardiac function and survival in response to myocardial ischemia. Circulation.

[28] Mao J.H., Sun X.Y., Liu J.X., Zhang Q.Y., Liu P., Huang Q.H., Li K.K., Chen Q., Chen Z., Chen S.J. (2010). As4S4 targets RING-type E3 ligase c-CBL to induce degradation of BCR-ABL in chronic myelogenous leukemia. Proc. Natl Acad Sci USA.

[29] Chang L., Kamata H., Solinas G., Luo J.L., Maeda S., Venuprasad K., Liu Y.C., Karin M. (2006). The E3 ubiquitin ligase itch couples JNK activation to TNFalpha-induced cell death by inducing c-FLIP(L)(L) turnover. Cell.

[30] Zhang G., Liu J., Zhang Y., Qu J., Xu L., Zheng H., Liu Y., Qu X. (2012). Cbl-b-dependent degradation of FLIP(L) is involved in ATO-induced autophagy in leukemic K562 and gastric cancer cells. FEBS Lett..

[31] Kleinesudeik L., Rohde K., Fulda S. (2018). Regulation of the antiapoptotic protein cFLIP by the glucocorticoid Dexamethasone in ALL cells. Oncotarget.

[32] Oh H.Y., Namkoong S., Lee S.J., Por E., Kim C.K., Billiar T.R., Han J.A., Ha K.S., Chung H.T., Kwon Y.G. (2006). Dexamethasone protects primary cultured hepatocytes from death receptor-mediated apoptosis by upregulation of cFLIP. Cell Death Differ..

[33] Lee T.J., Um H.J., Min do S., Park J.W., Choi K.S., Kwon T.K. (2009). Withaferin A sensitizes TRAIL-induced apoptosis through reactive oxygen species-mediated up-regulation of death receptor 5 and down-regulation of c-FLIP(L). Free Radic. Biol. Med..

[34] Woo S.M., Min K.J., Seo B.R., Kwon T.K. (2016). YM155 sensitizes TRAIL-induced apoptosis through cathepsin S-dependent down-regulation of Mcl-1 and NF-κB-mediated down-regulation of c-FLIP(L) expression in human renal carcinoma Caki cells. Oncotarget.

[35] Jang J.H., Kim E.A., Park H.J., Sung E.G., Song I.H., Kim J.Y., Woo C.H., Doh K.O., Kim K.H., Lee T.J. (2017). Methylglyoxal-induced apoptosis is dependent on the suppression of c-FLIP(L)(L) expression via down-regulation of p65 in endothelial cells. J. Cell. Mol. Med..

[36] Lang U.E., Kocabayoglu P., Cheng G.Z., Ghiassi-Nejad Z., Munoz U., Vetter D., Eckstein D.A., Hannivoort R.A., Walsh M.J., Friedman S.L. (2013). GSK3beta phosphorylation of the KLF6 tumor suppressor promotes its transactivation of p21. Oncogene.

[37] Kim S., Woo S.M., Min K.J., Seo S.U., Lee T.J., Kubatka P., Kim D.E., Kwon T.K. (2019). WP1130 Enhances TRAIL-induced apoptosis through USP9X-dependent miR-708-mediated downregulation of c-FLIP(L). Cancers (Basel).

[38] Min K.J., Shahriyar S.A., Kwon T.K. (2020). Arylquin 1, a potent Par-4 secretagogue, induces lysosomal membrane permeabilization-mediated non-apoptotic cell death in cancer cells. Toxicol. Res..

[39] Woo S.M., Seo S.U., Kubatka P., Min K.J., Kwon T.K. (2019). Honokiol Enhances TRAIL-Mediated Apoptosis through STAMBPL1-Induced Survivin and c-FLIP(L) Degradation. Biomolecules.

