# Supplementary material for: Dexamethasone Inhibits TRAIL-Induced Apoptosis through c-FLIP(L) Upregulation and DR5 Downregulation by GSK3β Activation in Cancer Cells
Source: Cancers (Basel). 2020 Oct 9;12(10):2901. doi: 10.3390/cancers12102901 (PMC7600459; doi:10.3390/cancers12102901)
Supplement: Supplementary file 1 [file cancers-12-02901-s001.pdf]

cancers

Supplementary Materials

Dexamethasone Inhibits TRAIL-Induced Apoptosis through c-FLIP(L) Upregulation and DR5 Downregulation by GSK3β activation in Cancer Cells

Mi-Yeon Jeon, Seon Min Woo, Seung Un Seo, Sang Hyun Kim, Ju-Ock Nam, Shin Kim, Jong-Wook Park, Peter Kubatka, Kyoung-jin Min, and Taeg Kyu Kwon

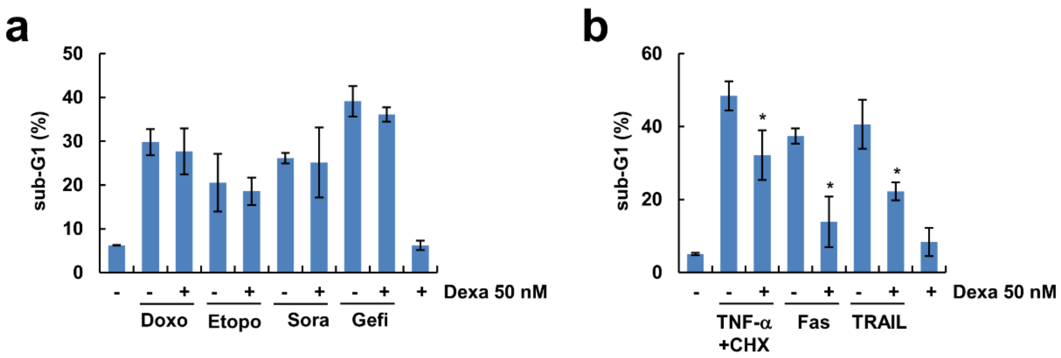

Figure S1. Effect of DEX on anti-cancer drugs-induced cell death. **(a,b)** Caki-1 cells were pretreated with the indicated concentrations of DEX for 1 h, and then treated with anti-cancer drugs (doxorubicin, etoposide, sorafenib and gefitinib) **(a)** or death receptor-associated drugs (TNF-α+CHX, Fas and TRAIL) **(b)** for 24 h. Apoptosis was determined by flow cytometry.

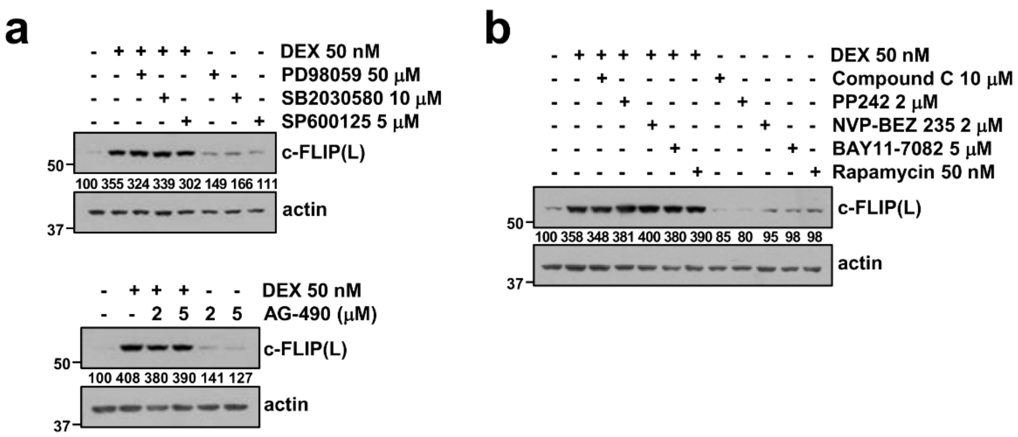

Figure S2. Effect of various multiple kinases inhibitors on DEX-mediated c-FLIP upregulation. **(a,b)** Caki-1 cells were pretreated with MAPKs inhibitors [PD98059 (MEK inhibitor), SB203580 (p38 MAPK inhibitor), and SP600125 (JNK inhibitor)], JAK/STAT inhibitor (AG-490) **(a)**, AMPK inhibitor (compound C), mTOR kinase inhibitors (PP242, NVP-BEZ235 and Rapamycin) and NF- B inhibitor (BAY11-7082) **(b)** for 1 h, and then treated with 50 nM DEX for 24 h. Protein expression was determined by western blotting.

Figure 1a

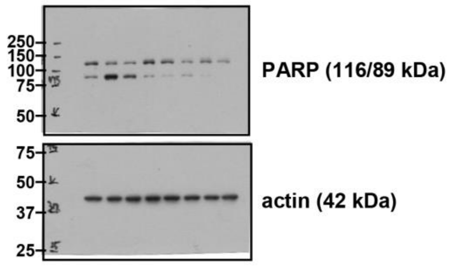

Figure 1d

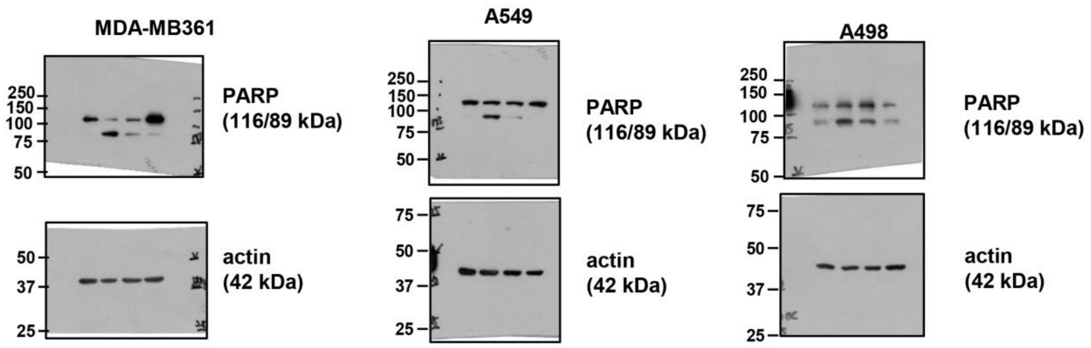

Figure S3. Uncropped western blots for Figure 1.

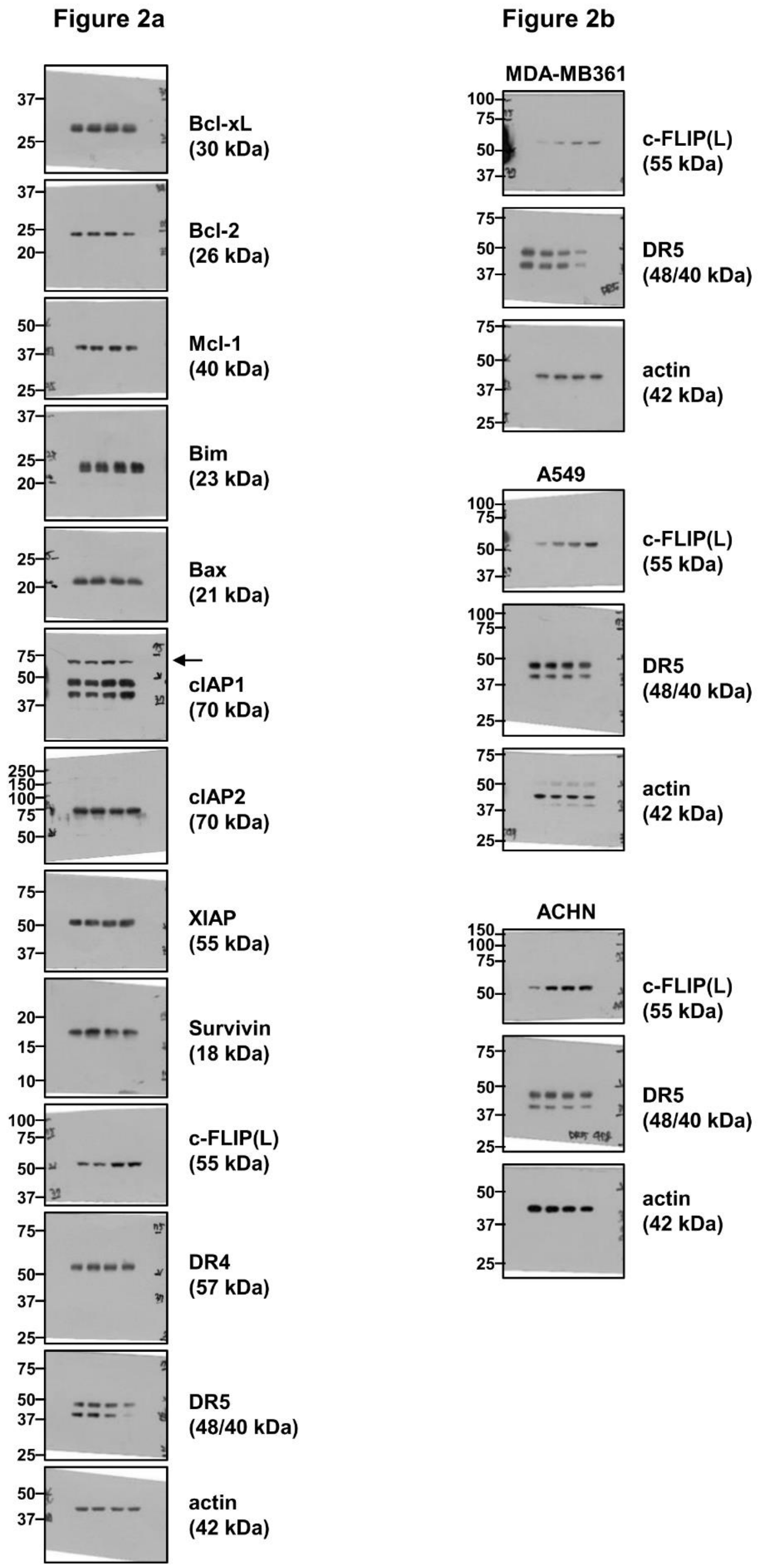

Figure S4. Uncropped western blots for Figure 2.

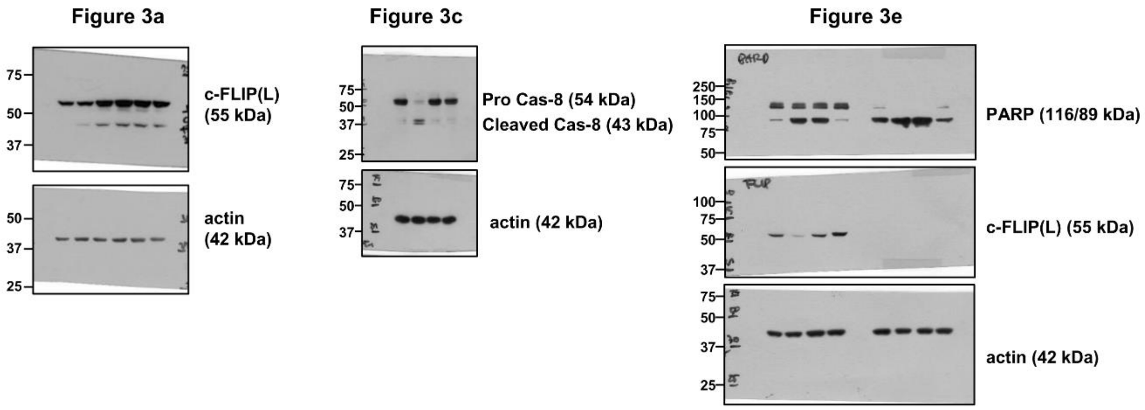

Figure S5. Uncropped western blots for Figure 3.

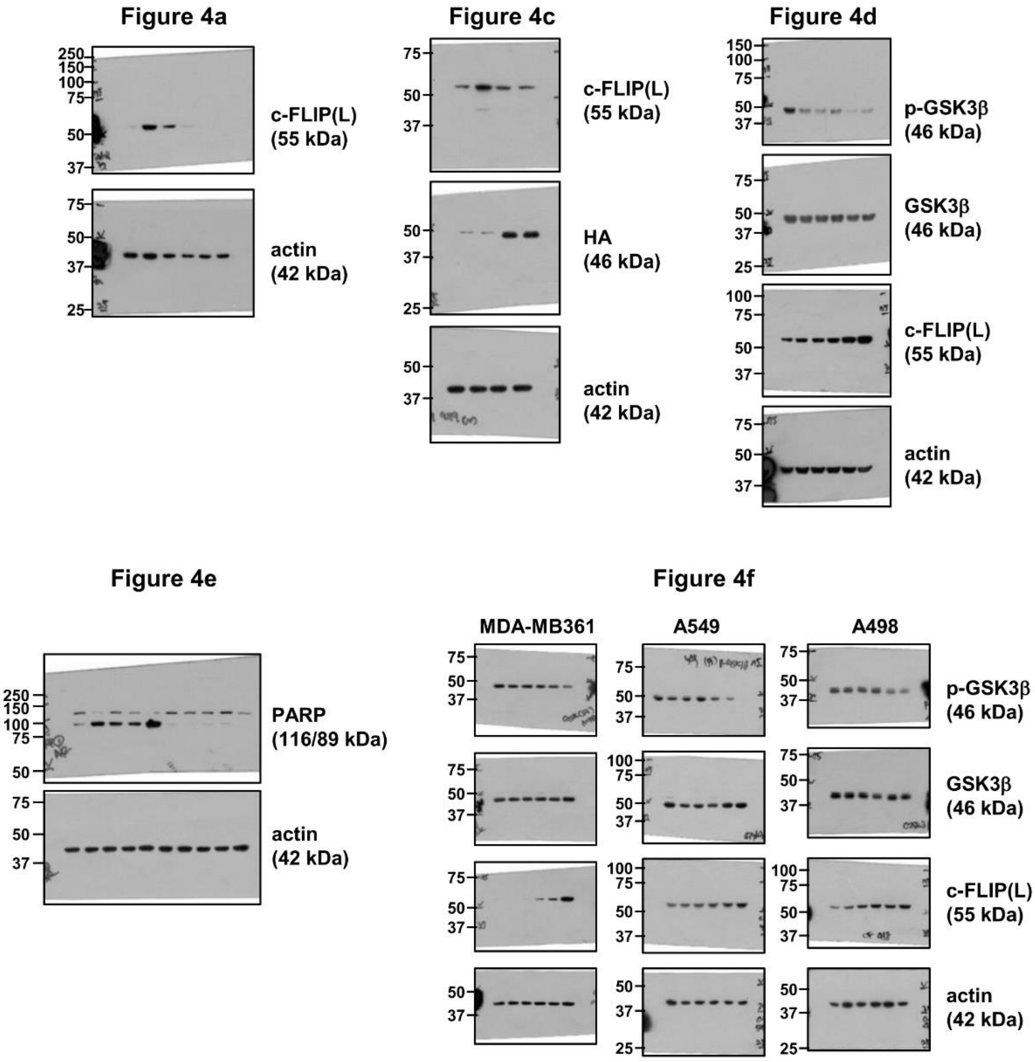

Figure S6. Uncropped western blots for Figure 4.

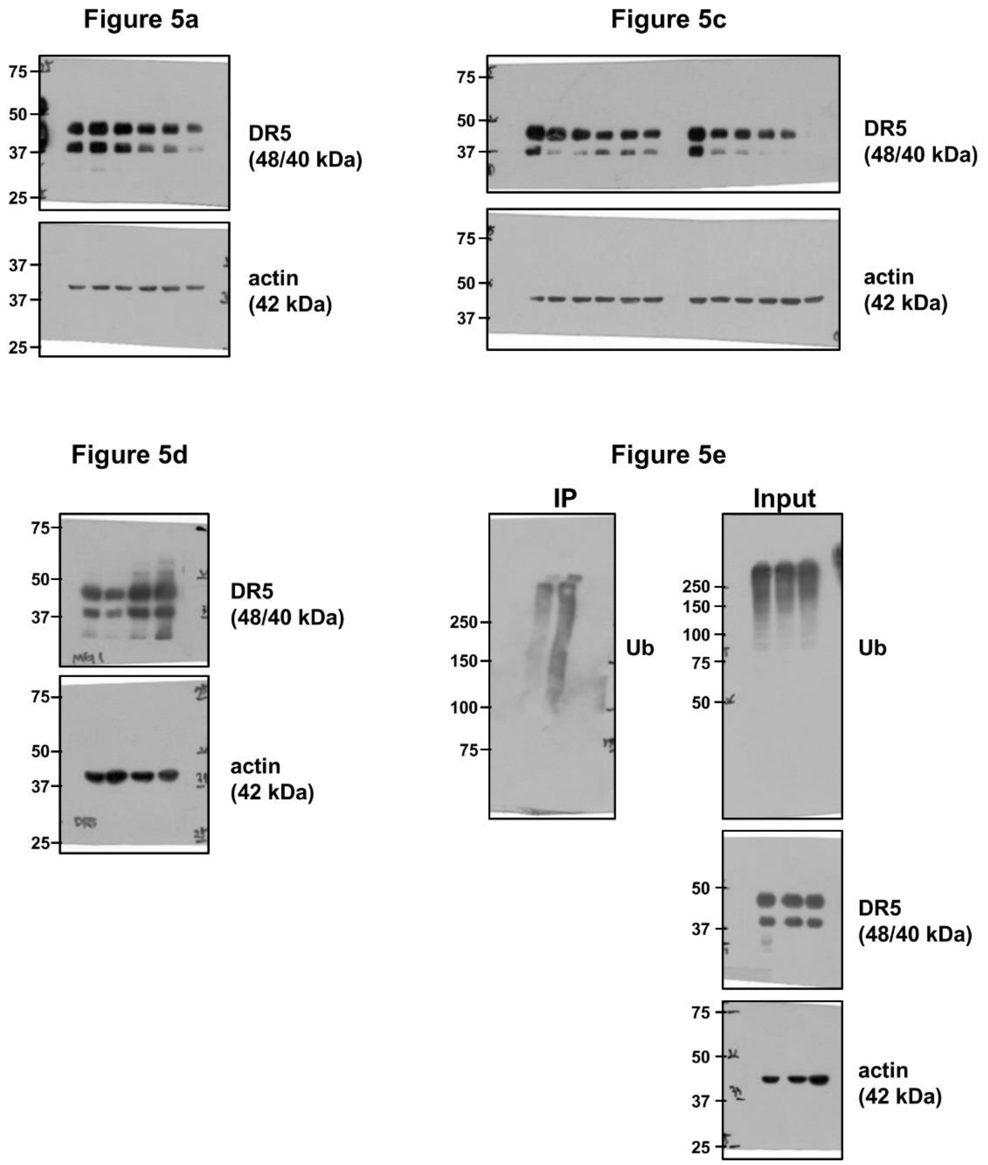

Figure S7. Uncropped western blots for Figure 5.

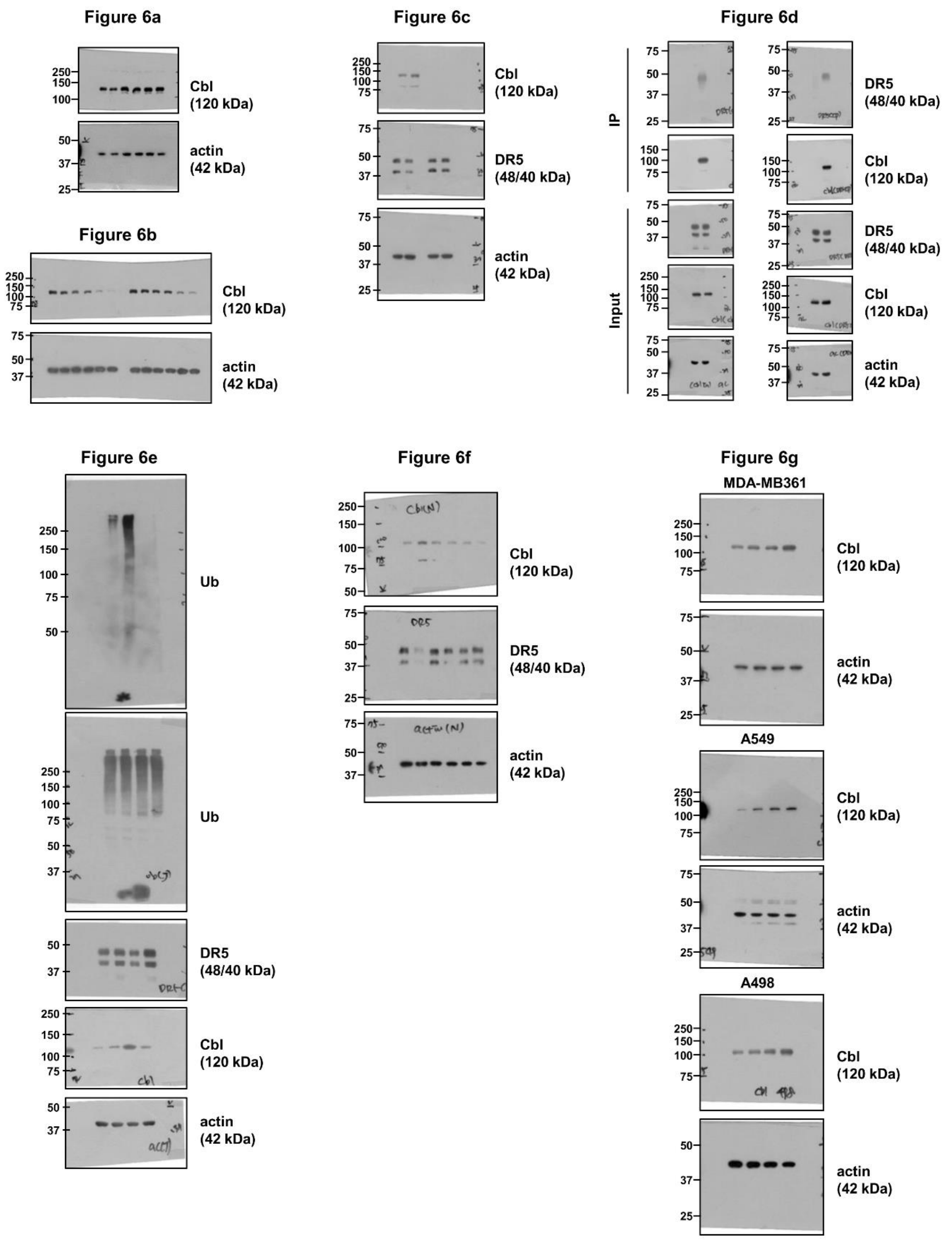

Figure S8. Uncropped western blots for Figure 6.

Supplemetary Figure 2a

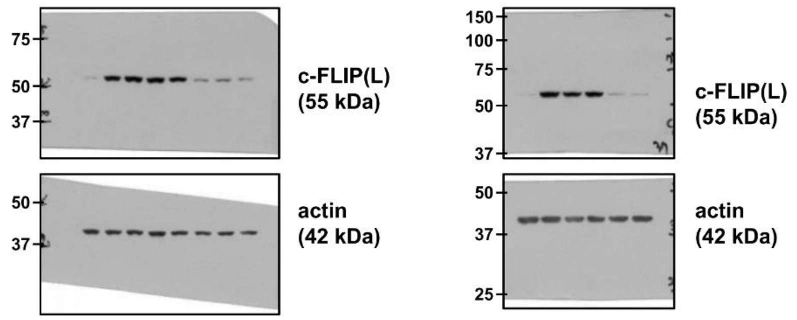

Supplemetary Figure 2b

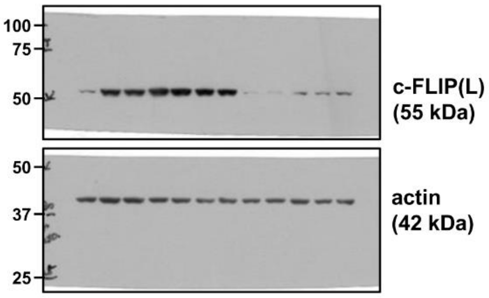

Figure S9. Uncropped western blots for Figure S2.

© 2020 by the authors. Submitted for possible open access publication under the terms and conditions of the Creative Commons Attribution (CC BY) license (http://creativecommons.org/licenses/by/4.0/).
